# Non-Mass Enhancements on DCE-MRI: Development and Validation of a Radiomics-Based Signature for Breast Cancer Diagnoses

**DOI:** 10.3389/fonc.2021.738330

**Published:** 2021-09-22

**Authors:** Yan Li, Zhenlu L. Yang, Wenzhi Z. Lv, Yanjin J. Qin, Caili L. Tang, Xu Yan, Yihao H. Guo, Liming M. Xia, Tao Ai

**Affiliations:** ^1^Department of Radiology, Tongji Hospital, Tongji Medical College, Huazhong University of Science and Technology, Wuhan, China; ^2^Department of Artificial Intelligence, Julei Technology Company, Wuhan, China; ^3^Scientific Marketing, Siemens Healthcare Ltd., Shanghai, China; ^4^Magnetic Resonance (MR) Collaboration, Siemens Healthcare, Guangzhou, China

**Keywords:** breast cancer, non-mass enhancement, radiomics, differential diagnosis, magnetic resonance imaging

## Abstract

**Purpose:**

We aimed to assess the additional value of a radiomics-based signature for distinguishing between benign and malignant non-mass enhancement lesions (NMEs) on dynamic contrast-enhanced breast magnetic resonance imaging (breast DCE-MRI).

**Methods:**

In this retrospective study, 232 patients with 247 histopathologically confirmed NMEs (malignant: 191; benign: 56) were enrolled from December 2017 to October 2020 as a primary cohort to develop the discriminative models. Radiomic features were extracted from one post-contrast phase (around 90s after contrast injection) of breast DCE-MRI images. The least absolute shrinkage and selection operator (LASSO) regression model was adapted to select features and construct the radiomics-based signature. Based on clinical and routine MR features, radiomics features, and combined information, three discriminative models were built using multivariable logistic regression analyses. In addition, an independent cohort of 72 patients with 72 NMEs (malignant: 50; benign: 22) was collected from November 2020 to April 2021 for the validation of the three discriminative models. Finally, the combined model was assessed using nomogram and decision curve analyses.

**Results:**

The routine MR model with two selected features of the time-intensity curve (TIC) type and MR-reported axillary lymph node (ALN) status showed a high sensitivity of 0.942 (95%CI, 0.906 - 0.974) and low specificity of 0.589 (95%CI, 0.464 - 0.714). The radiomics model with six selected features was significantly correlated with malignancy (P<0.001 for both primary and validation cohorts). Finally, the individual combined model, which contained factors including TIC types and radiomics signatures, showed good discrimination, with an acceptable sensitivity of 0.869 (95%CI, 0.816 to 0.916), improved specificity of 0.839 (95%CI, 0.750 to 0.929). The nomogram was applied to the validation cohort, reaching good discrimination, with a sensitivity of 0.820 (95%CI, 0.700 to 0.920), specificity of 0.864 (95%CI,0.682 to 1.000). The combined model was clinically helpful, as demonstrated by decision curve analysis.

**Conclusions:**

Our study added radiomics signatures into a conventional clinical model and developed a radiomics nomogram including radiomics signatures and TIC types. This radiomics model could be used to differentiate benign from malignant NMEs in patients with suspicious lesions on breast MRI.

## 1 Introduction

According to the American College of Radiology (ACR) BI-RADS^®^ Atlas, 5th edition (1), breast lesions with abnormal enhancement variables on dynamic contrast-enhanced breast magnetic resonance imaging (breast DCE-MRI) include foci, masses, and non-mass enhancement lesions (NMEs). In 2020, breast cancer became the most common cancer of women worldwide (2), and the differentiation between benign and malignant breast lesions using MRI-based diagnostics was found to be critical for breast cancer treatments. However, distinguishing benign and malignant breast lesions on DCE-MRI is challenging, especially when NMEs are present (3).

NMEs are associated with a wide-ranging spectrum of different pathologic findings (4–6), with an overlap in the imaging findings between malignant and benign lesions. NMEs remain a diagnostic challenge for radiologists despite the frequent attempts to distinguish benign from malignant NMEs using different methodologies, including conventional morphologic comparisons (6–8) and the measurement of different parameters, such as ADC values and the initial slope of kinetic curves (9–11). Baltzer et al. reported that the primary cause for false positive results of breast MRI may due to NMEs, resulting in unnecessary biopsies (12). Studies have shown that morphologic assessments are disputable in attempting to differentiate benign *vs*. malignant NMEs. Some studies have demonstrated that morphologic assessments are more useful than kinetic assessments in distinguishing NMEs (13–15), while other studies have reported that morphologic assessments have a relatively low specificity and sensitivity to distinguish NMEs (16–18). In addition, morphologic assessments depend on the human eye are subjective with limitations; thus, substantial inter- and intra-observer variability is seen with these assessments (19). A meta-analysis (20) showed heterogeneity among studies with sensitivities from 0% to 100% and specificities from 48% to 100%. These factors underscore the complexity of the diagnostic phase and simultaneously present a therapeutic challenge. For example, idiopathic granulomatous mastitis, a benign inflammatory disease, can mimic breast cancer, both clinically and radiologically (21, 22).

In recent years, radiomics, a technology of transforming digital medical images into quantifiable data to improve medical decisions (23), has been found to have a potential benefit in increasing the knowledge base of diagnostic oncology and predicting the accuracy of medical imaging. Radiomics is partially based on the hypothesis that medical images contain much more information than can be visually deciphered by radiologists (24). According to our best knowledge, there is little research reported the additional value of radiomics to differentiate benign *vs*. malignant NMEs on DCE-MRI. Additionally, to date, a model that combines a radiomics signature and conventional analysis to produce superior diagnostic performance in diagnosing malignant NMEs has yet to be reported.

In this study, we developed and validated a nomogram that combined radiomics and conventional analytic clinical factors to evaluate the additional value of radiomics in differentiating benign from malignant NMEs. We also compared the diagnostic performance of the nomogram with the radiomics score and analytic clinical factors alone.

## 2 Materials and Methods

### 2.1 Patients

**Figure 1:**
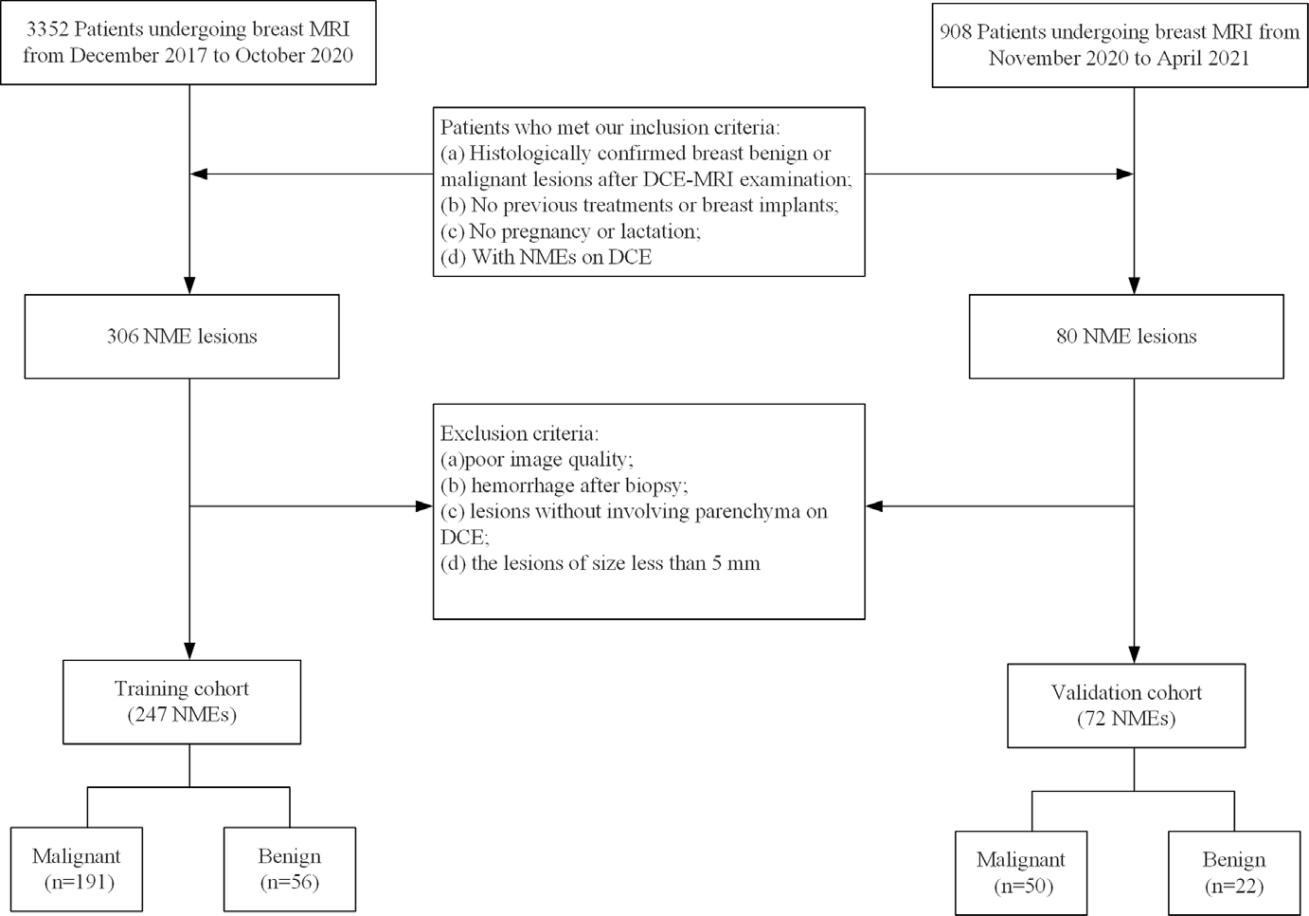
Flowchart of the study population enrollment. NME, non-mass enhancement lesion.

We retrospectively reviewed 3352 consecutive patients who underwent breast MRI in our hospital between December 2017 and October 2020. In total, 232 female patients with 247 lesions were selected and comprised the primary training cohort (mean age, 44.8 ± 10.6 years). Among these patients, 14 had additional lesions in the contralateral breast and 1 patient had two lesions in different quadrants of her left breast. The inclusion criteria were as follows: (a) histologically confirmed benign or malignant breast lesions on DCE-MRI examinations; (b) no previous treatments or breast implants; (c) no pregnancy or lactation; and (d) NMEs found on DCE images. Patients were excluded if image quality was poor, hemorrhage was present after biopsy, lesions did not involve parenchyma on the DCE images, or the lesion sizes were <5 mm. Using this inclusion and exclusion criteria, a validation cohort of 72 consecutive female patients (mean age, 47.9± 11.2 years) was selected from 908 consecutive patients between November 2020 and April 2021 in our hospital. A flowchart of this study is presented in **Figure 1**. For each patient, conventional clinical data, including age and menopause status, were obtained from electronic medical records.

### 2.2 Magnetic Resonance Image Acquisition

MR examinations for both the validation cohort and training cohort were obtained on a 3T scanner (MAGNETOM Skyra, Siemens Healthcare, Erlangen, Germany) in our hospital. All scans were performed with a dedicated 16-channel phased-array breast coil in the prone position using the same protocol.

For breast diffusion-weighted imaging (DWI), multi-b-value DWI was applied with a readout-segmented technique (RESOLVE DWI), similar to our previous works (25): repetition time (TR) = 5000 ms, echo time (TE) = 70 ms, field of view (FOV) = 169 x 280 mm^2^, matrix size = 114 x 188, slice thickness = 5.0 mm, readout segment = 5, average = 1, diffusion gradient mode = 3-scan-trace, b values = 0, 50, 1000s/mm^2^, and acquisition time = 4:27 (min: sec).

For breast DCE-MRI, a protocol based on time-resolved angiography was used with a stochastic trajectory, volume-interpolated breath-hold examination sequence (TWIST-VIBE). The detailed scan parameters were as follows: TR = 5.24 ms, TE = 2.46 ms, matrix size = 182 x 320, FOV = 260 x 320 mm^2^, slice thickness = 1.5 mm without gap, flip angle = 10°, temporal resolution = 5.74 s/phase, and acquisition time = 5:57(min: sec).

The contrast medium (Omniscan, GE Healthcare, Milwaukee, WI) was intravenously injected with a power injector at the end of the third acquisition phase. The dose was 0.1 mmol/kg body weight, with an injection rate of 2.5 mL/s, which was followed by a 20 mL saline flush.

### 2.3 Image Interpretation

For each patient in the training cohort and the validation cohort, two radiologists (Y.L. and T.A. with 8 and 10 years of experience in breast MRI, respectively), were blinded to the pathologic results. Each radiologist reviewed all breast MR images from the 304 patients, assessing breast density, the degree of background parenchymal enhancement, and MR-reported lymph node status by consensus. The maximal diameter, internal enhancement, and distribution were recorded in the very early phase (about 90 seconds) after contrast media injection according to the BI-RADS 5^th^ edition (1). Of these, the maximal diameter was assessed on multiplanar reformatted images using a Siemens clinical workstation. The type of time-intensity curve (TIC) for each case was drawn based on DCE-MRI with a region of interest (ROI) of approximately 0.2-0.4 cm^2^ placed on each slice at the brightest part of the lesions on images obtained in the early phase after the contrast injection. We recorded the high-level TIC curve types when different types were present in each lesion. On all slices of the apparent diffusion coefficient (ADC) maps, multiple ROIs were carefully placed on the darkest areas, which were confirmed by agreement by the two radiologists. Thus, the lowest ROI ADC value was regarded as the minimum ADC value for each lesion. If no lesions could be evaluated with DWI or the ADC maps, we copied ROIs on the DCE-MRI image and pasted them on the ADC maps. We defined the axillary lymph node (ALN) with a maximal short diameter of ≥10mm, an absent fatty hilum, or a long axis/short axis of <2 as MR-reported ALN positive. Vasodilation of the surrounding feeding artery was defined as positive on maximum intensity projection images (MIPs) and was included based on our experience. The above-mentioned factors were all initial clinical candidate predictors for NME differentiation.

### 2.4 Features Extraction and Radiomics Signature

The radiomics signature was applied to the clinical analyses, and a diagnostic model for differentiation was developed using the training cohort. The radiomics analysis was performed on the very early phase (90 seconds) images after contrast media injection, as was the morphologic evaluation. Prior to the radiomics analysis, the images of each case were transferred into the open-source software, ITK-SNAP (Version 3.8.0), to perform semi-automatically ROI segmentation. ROIs were drawn with care to include the whole lesion, avoiding normal glandular tissue, fat, vessels, and necrosis. Pyradiomics open-source software (https://pyradiomics.readthedocs.io/en/latest/index.html) was used to automatically extract tissue intensities and textural, morphologic, and wavelet features. We used the least absolute shrinkage and selection operator (LASSO) method, an appropriate tool for high-dimensional data regression (26), to select the most effective features from the training cohort data set. For each lesion, a radiomics score (Rad-score) was calculated weighting by the respective coefficients of selected features.

### 2.5 Nomogram in the Training Cohort and Validation

Initial clinical multivariate logistic regression analysis included age, menopause status, maximal diameter, fibrotic gland tissue, background parenchymal enhancement, morphologic assessment, ALN status, and TIC assessment on DCE-MRI and the minimum ADC values on DWI. We added radiomics features into the clinical multivariable logistic regression analysis and built the radiomics nomogram to supply the radiologists and clinicians with an effective tool for differentiating benign and malignant NMEs. The calibration curve and Hosmer & Lemeshow test (27) were adapted to evaluate the radiomics nomogram calibration. Nomogram performance was evaluated using the area under the curve (AUC) analysis.

#### 2.5.1 Consistency Validation

In the data set of the training cohort, consistency validation was performed by comparing the first measurement and second measurement one month later of reader 1 (Y.L.) for intra-observer agreement. The second measurement of reader 1 and the extraction of reader 2 (Z.L.Y) in 60 patients were compared to produce inter-observer agreement. The interclass correlation coefficient (ICC) was applied to assess the feature extraction agreement, which was greater than 0.80 and considered excellent.

#### 2.5.2 Data Validation

We applied the same method as that of the training cohort to calculate the Rad-score in the validation cohort. We applied the logistic regression equation produced in the training cohort to all lesions of the validation cohort. We tested the performance of the nomogram using calibration and AUC analyses.

### 2.6 Statistical Analysis

R (RStudio, Version 3.6.3) software was used for algorithms and statistical analyses. For continuous variates, Student’s t-tests were performed. For categorical variates, the chi-square test or Wilcoxon rank-sum test were applied. We used univariate logistic regression analysis to determine potential factors affecting differentiation. Then, logistic regression models containing the above-mentioned potential factors were used for multivariate analysis. A nomogram was built on the logistic regression model as a graphical presentation. The area under the receive operating characteristic (AUC-ROC) curve, accuracy, sensitivity, and specificity were applied to indicate the discriminative ability of each factor and nomogram. P-values <0.05 (two-tailed) was considered statistically significant.

## 3 Results

### 3.1 Conventional Clinical Analysis

#### 3.1.1 Training Cohort

In the training cohort, of the 247 lesions, 191 malignant and 56 benign lesions were confirmed pathologically by either biopsy, lumpectomy, or mastectomy. For the patient who had two lesions in the left breast, the lesion in the upper outer quadrant was confirmed as adenosis, while the lesion in the medial area was ductal cancer *in situ*. Specific pathologic results are shown in **Table 1**. Internal enhancement patterns, background parenchymal enhancements (BPEs), and MRI reported-fibroglandular tissue (FGT) were not different between malignant and benign lesions (P=0.397, 0.760, 0.139). The mean age of the patients with malignant lesions was older than that of the benign cases (P=0.035). The maximal diameter of the malignant lesions was significantly longer than that of the benign lesions (P<0.001). A higher proportion of postmenopausal women were found in the malignant group than in the benign group (P=0.034). The constituent ratio of distribution was significantly different between malignant and benign cases (P<0.001). Of these, the proportion with linear distributions was higher in the benign group than in the malignant group (P=0.046). The minimum ADC value of the malignant lesions was significantly lower than that of the benign lesions (P<0.001). The malignant group had a significantly higher percentage of higher-level TIC pattern types and MR-reported ALN-positive and MIP-positive cases (all P<0.001). Specific results are shown in **Table 2**. Age, menopause status, maximal diameters, distributions, TIC patterns, minimum ADC values, MRI reported-ALN status, and MIP status were potential factors influencing differentiation according to the univariate logistic regression analysis. From the multivariate analysis results, higher-level TIC pattern types, and MR-reported ALN-positive statuses were significantly associated with malignancy (all P<0.001). The AUCs, sensitivities, and specificities of the clinical multivariate regression model developed using TIC types and MR-reported ALN status were 0.852 (95%CI: 0.799-0.906), 0.942 (95%CI: 0.906-0.974), and 0.589 (95%CI:0.446-0.714), respectively, to differentiate between malignant and benign NME lesions. The specific results are shown in **Table 3**.

**Table 1:** Pathologic findings for all non-mass enhancement (NME) lesions.

Pathological results	Training Cohort(n = 247)	Validation Cohort(n = 72)
**Benign**	56	22
Adenosis	49	22
Papilloma	1	0
Chronic inflammation	5	0
Fibroadenoma/fibroadenomatous change	1	0
**Malignant**	191	50
IDC	88	21
ILC	11	0
Pure DCIS	31	9
Invasive cancer with CIS	50	14
CIS with invasive component	24	6
Mucinous carcinoma	1	0

IDC, Invasive ductal carcinoma; ILC, Invasive lobular carcinoma; DCIS, ductal carcinoma in situ; CIS, cancer in situ.

**Table 2:** Characteristics of patients in the training and validation cohorts.

Characteristic	Training Cohort			Validation Cohort		
	Malignant (n = 191)	Benign (n = 56)	P	Malignant (n = 50)	Benign (n = 22)	P
**Age**, mean ± SD, years	45.4 ± 10.2	42.0 ± 11.8	0.035	51.4 ± 10.2	39.9 ± 9.5	<0.001
**Menopause status**, No (%)						
Postmenopausal	41 (21.5)	5 (8.9)	0.034	20 (40)	2 (9.1)	0.011
premenopausal	150 (78.5)	51 (91.1)		30 (60)	20 (90.9)	
**MRI reported-FGT**, No (%)						
a	1 (0.5)	0 (0)		2 (4)	1 (4.5)	
b	37 (19.4)	8 (14.3)		11 (22)	3 (13.6)	
c	140 (73.3)	44 (78.6)		34 (68)	15 (68.1)	
d	13 (6.8%)	4 (7.1)	0.395	3 (6)	3 (13.6)	0.331
**MRI reported-BPE**, No (%)						
Minimal-Mild	143 (74.9)	36 (64.3)		24 (48)	7 (31.8)	
Moderate	43 (22.5)	19 (33.9)		23 (46)	10 (45.5)	
Marked	5 (2.6)	1 (1.8)	0.099	3 (6)	5 (22.7)	0.079
**Maximal diameter**, mean ± SD, mm	47.7 ± 21.4	32.9 ± 18.9	<0.001	44.3 ± 16.6	29.6 ± 10.6	<0.001
**NME Enhancement patterns**						
**Distribution**, No (%)						
Focal	21 (11.0)	16 (28.6)		0 (0)	0 (0)	
Linear	2 (1.0)	6 (10.7)		0 (0)	4 (18.2)	
Segmental	38 (19.9)	11 (19.6)		13 (26)	5 (22.7)	
Regional	82 (42.9)	14 (25.0)		26 (52)	13 (59.1)	
Multiple regions	35 (18.3)	8 (14.3)		9 (18)	0 (0)	
Diffuse	13 (6.8)	1 (1.8)	<0.001	2 (4)	0 (0)	0.016
**Internal enhancement patterns**, No (%)						
Homogeneous	11 (5.8)	7 (12.5)		2 (4)	4 (18.2)	
Heterogeneous	127 (66.5)	34 (60.7)		36 (72)	12 (54.5)	
Clumped	46 (24.1)	13 (23.2)		9 (18)	6 (27.3)	
Clustered ring	7 (3.7)	2 (3.6)	0.438	3 (6)	0 (0)	0.474
**TIC pattern**, No (%)						
Persistent	11 (5.8)	33 (58.9)		3 (6)	12 (54.5)	
Plateau	82 (42.9)	18 (32.1)		27 (54)	9 (40.9)	
Washout	98 (51.3)	5 (8.9)	<0.001	20 (40)	1 (4.5)	<0.001
**Minimum ADC value**, mean ± SD, 10^-6 mm2/s	769.3 ± 173.4	914.2 ± 247.8	<0.001	730.1 ± 147.8	898.5 ± 118.1	<0.001
**MRI reported- ALN status**, No (%)						
ALN-positive	70 (36.6)	4 (7.1)		15 (30)	0 (0)	
ALN-negative	121 (63.4)	52 (92.9)	<0.001	35 (70)	22 (100)	0.003
**MIP**						
positive	117 (61.3)	17 (30.4)		16 (32)	8 (36.4)	
negative	74 (38.7)	39 (69.6)	<0.001	34 (68)	14 (63.6)	0.789
**Radiomics score**, median (interquartile range)	1.833 (1.320to 2.391)	0.368 (-0.335 to 0.977)	<0.001	1.453 (1.108 to 2.074)	0.376 (-0.161 to 0.925)	<0.001

Percentages may not add up to 100 because of rounding. TIC, Time Intensity Curve; BPE, Background Parenchymal Enhancement; FGT, Fibro glandular Tissue; ALN, Axillary Lymph Nodes; MIP, Maximum Intensity Projection.

**Table 3:** Risk factors for malignancy and the performance of the clinical and combined models for breast non-mass enhanced (NME) lesions.

Intercept and Variable	Clinical model			Radiomics model	Combined model		
	β	Odds Ratio (95% CI)	P	NA	β	Odds Ratio (95% CI)	P
**Intercept**	-3.064		<0.001	NA	-3.167		<0.001
**TIC types**	2.049	7.761 (4.225 to14.259)	<0.001	NA	1.463	4.319 (2.310 to 8.074)	<0.001
**MR-reported ALN status**	1.399	4.052 (1.262 to13.009)	0.019	NA	0.680	1.975 (0.526 to 7.419)	0.314
**Radiomics signature**	NA	NA	NA	NA	1.173	3.233 (1.963 to 5.325)	<0.001
**AUC**							
Training cohort		0.852 (0.798 to 0.906)		0.864 (0.805 to 0.923)		0.908 (0.864 to 0.952)	
Validation cohort		0.842 (0.758 to 0.926)		0.876 (0.791 to 0.962)		0.901 (0.827 to 0.974)	
**Sensitivity**							
Training cohort		0.942 (0.906 to 0.974)		0.827 (0.770 to 0.880)		0.896 (0.817 to 0.916)	
Validation cohort		0.940 (0.860 to 1.000)		0.800 (0.680 to 0.900)		0.820 (0.700 to 0.920)	
**Specificity**							
Training cohort		0.589 (0.464 to 0.714)		0.804 (0.696 to 0.911)		0.839 (0.750 to 0.862)	
Validation cohort		0.545 (0.364 to 0.727)		0.863 (0.727 to 1.000)		0.864 (0.682 to 1.000)	

β is the regression coefficient; NA, not applicable; TIC, time-intensity curve; ALN, axillary lymph node; AUC, area under the curve.

#### 3.1.2 Validation Cohort

In the validation dataset, there were 50 malignant lesions and 22 benign lesions. Like the training dataset, internal enhancement patterns, MRI reported-FGT, and BPE were not significantly different between malignant and benign lesions. Moreover, no significant differences were found between the two cohorts regarding the MIP status. When applying the clinical multivariate logistic regression equation of the primary cohort to the validation dataset, the AUCs, sensitivities, and specificities were 0.842(95%CI: 0.758-0.926), 0.940(95%CI: 0.860-1.000), and 0.545(95%CI: 0.364-0.727), respectively (**Table 3**).

### 3.2 Radiomics Analysis and the Combined Model

#### 3.2.1 Training Cohort

**Figure 2:**
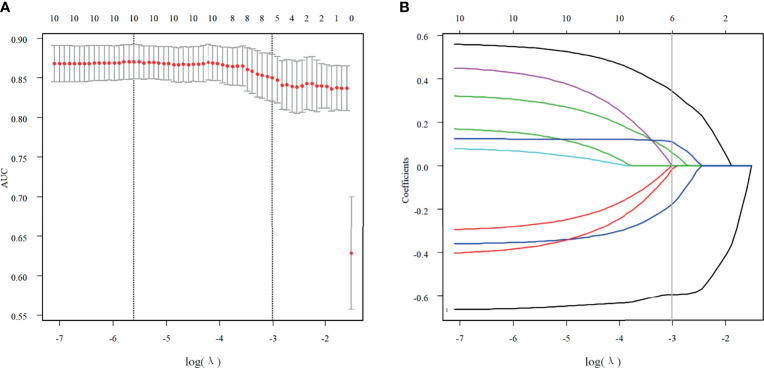
Texture feature selection. **(A)** Using the LASSO model, tuning parameter (λ) selection was according to a 5-fold cross-validation. Using the minimum criteria and the 1 standard error of the minimum criteria, dotted vertical lines were drawn for the optimal values. A λ value of 0.0495 with a log (λ) of -3.005783 was chosen for the 5-fold cross-validation. **(B)** According to the log (λ) sequence, a coefficient profile plot was produced. At the value selected with the 5-fold cross-validation, a vertical line was drawn, where the optimal λ resulted in six non-zero coefficients.

Of all features extracted from the lesions in the primary cohort, six features were selected as potentially effective factors for differentiation and were applied in the Rad-score calculation (**Figure 2**). The final computation of the model coefficients led to the following differentiation model for NMEs:


Rad−score = −0.594(original_shape_SurfaceVolumeRatio)+0.061(wavelet.HLL_glcm_ldn)−0.176(original_firstorder_Skewness)+0.343(wavelet.LLH_glcm_ldmn)−0.017(original_glszm_SmallAreaEmphasis)+0.110(wavelet.LLL_firstorder_Kurtosis)+1.468

**Figure 3:**
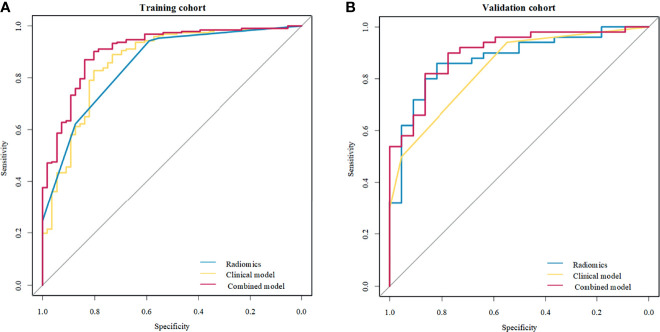
Receiver operating characteristic (ROC) curves of the clinical model, radiomics signature, and combined model to differentiate benign from malignant non-mass enhancement (NME) lesions. **(A)** Three methods in the training cohort; **(B)** Three methods in the validation cohort.

**Figure 4:**
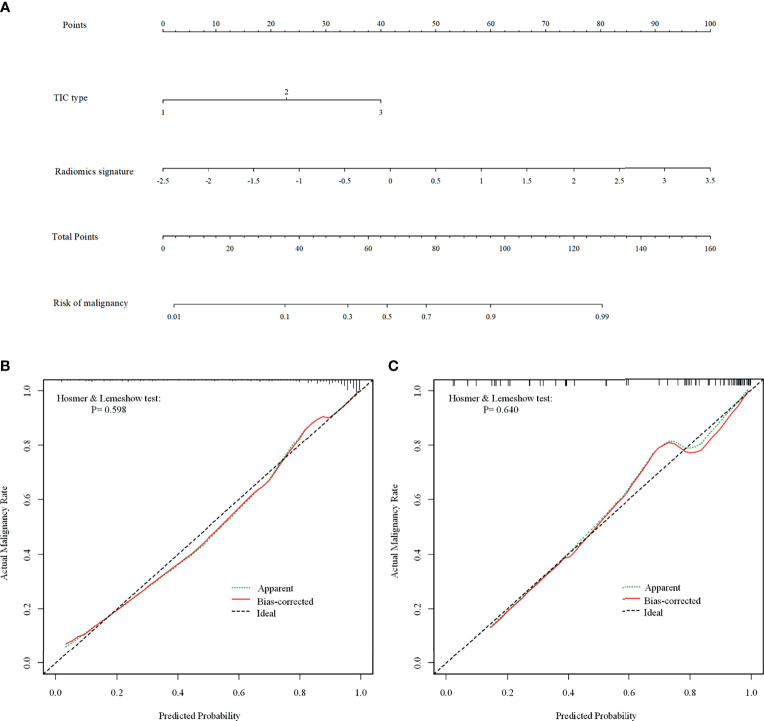
The combined nomogram for differentiating benign and malignant non-mass enhancement (NME) lesions. **(A)** The radiomics nomogram developed with the training cohort included time-intensity curve (TIC) types and radiomics signatures. **(B, C)** Calibration curves of the combined model in the training **(B)** and validation **(C)** cohorts. The Bias-corrected line represents the nomogram performance. The closer the red Bias-corrected line is to the diagonal dotted (ideal) line indicates a better differentiation performance.

Of the six features, the biggest weight was given to the shape feature (Surface Area to Volume Ratio). A significant difference in the Rad-score between benign and malignant NMEs was found in the training cohort (P<0.001). The AUC, sensitivity, and specificity of the radiomics multivariable logistic regression alone for NME differentiation was 0.864 (95%CI: 0.805-0.923), 0.827 (95%CI: 0.770-0.880), and 0.804 (95%CI: 0.696-0.893) (**Figure 3**, **Table 3**). After adding the radiomics analysis into the clinical multivariate regression model, MR-reported ALN status was no longer an independent factor of malignancy. We built a nomogram for the training cohort based on the TIC types and the radiomics signature (**Figure 4**), the specificity of which was improved from 0.589 (95%CI: 0.464- 0.714) in the clinical model to 0.839 (95%CI: 0.750- 0.862) in the combined model (**Table 3**). The final regression equation and correlation coefficients were calculated. In **Table 3**, the parameters in detail are reported. Using ROC curve analysis, the optimal cutoff value of the final regression equation was 0.772. Lesions with values below the cutoff value are judged as benign, while those with values exceeding the cutoff value are judged as malignant.

#### 3.2.2 Validation Cohort

In the validation cohort, there was also a significant difference in the Rad-score between benign and malignant NMEs (P<0.001). After adding the Rad-score analysis into the clinical model, the specificity increased from 0.545 (95%CI: 0.364- 0.727) to 0.864 (95%CI: 0.682- 1.000) (**Table 3**).

**Figure 5:**
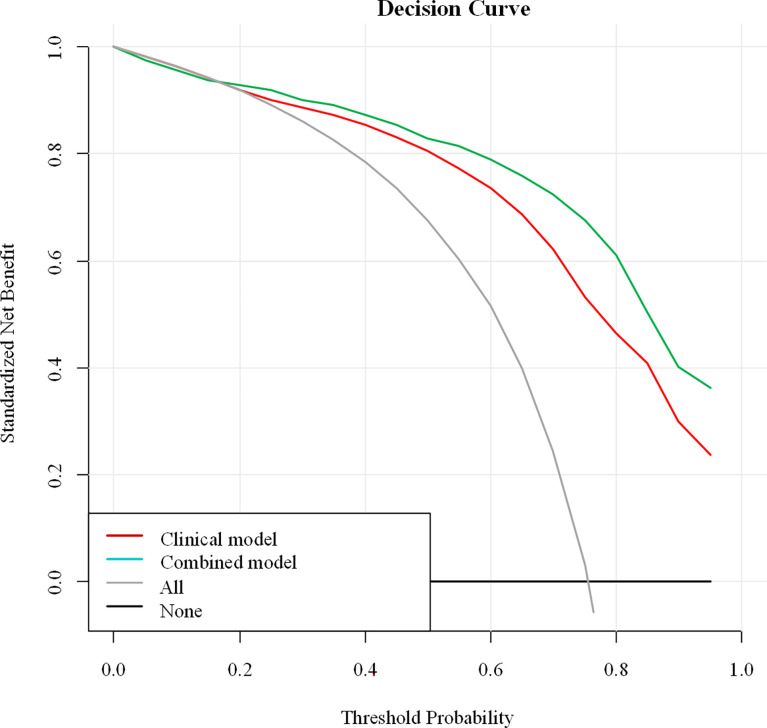
Decision curve analysis of the combined model. The Y-axis demonstrates the net benefit to patients. As indicated in the curve, the net benefit of using the combined model to differentiate benign and malignant NME lesions is greater than when the clinical model is used at a threshold probability of > 0.19.

For the differentiation between benign and malignant NMEs, the calibration curve of the combined model demonstrated excellent agreement between the prediction and real pathologic results in the training cohort as well as the validation cohort (**Figure 4**). In clinical medicine, the decision curve analysis for the combined model was developed according to a previous study (28) and is showed in **Figure 5**. The decision curve demonstrated that if the threshold probability was >19%, the nomogram could add more benefit to the discrimination of benign and malignant NMEs than the clinical model.

### 3.3 Consistency Validation

Based on the comparisons of radiomics feature measurements assessed one month apart by reader 1, the intra-observer agreement was excellent (ICC value=0.936, 95%CI: 0.929 to 0.942). Using the second measurements of the 60 patients assessed by reader 1 and the features extraction of the same data set assessed by reader 2, inter-observer was also excellent (ICC value =0.887, 95%CI: 0.876 to 0.898).

**Figure 6:**
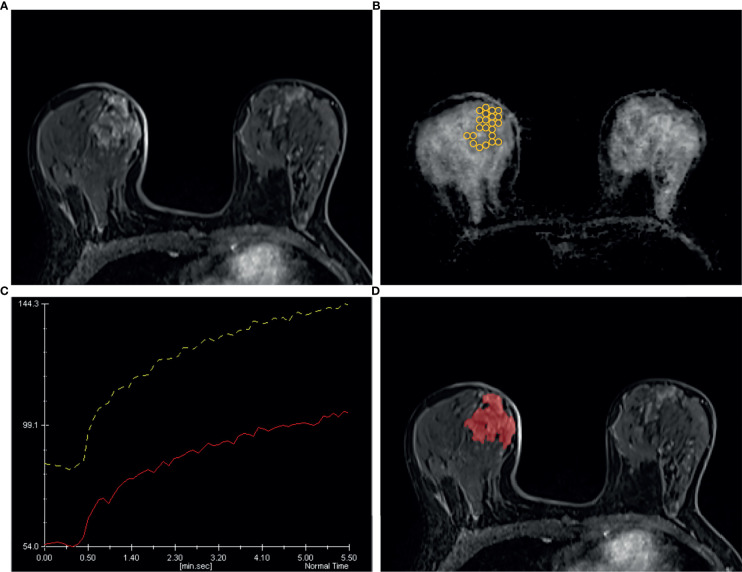
A 49 years old woman diagnosed as BIRADS 4 preoperatively by radiologists and confirmed as adenosis by operation. **(A)** Axial dynamic contrast-enhancement images obtained in the very early phase (about 90 seconds) show a non-mass enhancement lesion with segmental distribution in the right breast. **(B)** On the ADC map, multiple ROIs are placed to cover the whole area of the lesion. The ADC map shows the minimum ADC value of the ROIs is 1056 ×10^−6^ mm^2^/s. **(C)** After drawing the TIC curves for all ROIs at the brightest part on each slice, the high-level TIC curve type of this lesion is persistent type. **(D)** Using the ITK-SNAP software, the whole lesion was segmented. Finally, the logistic regression equation of the combined model for this lesion was calculated as 0.669, which was lower than the cut-off value 0.772 and adjudicated as benign lesion, consistent with the pathological results.

**Figure 7:**
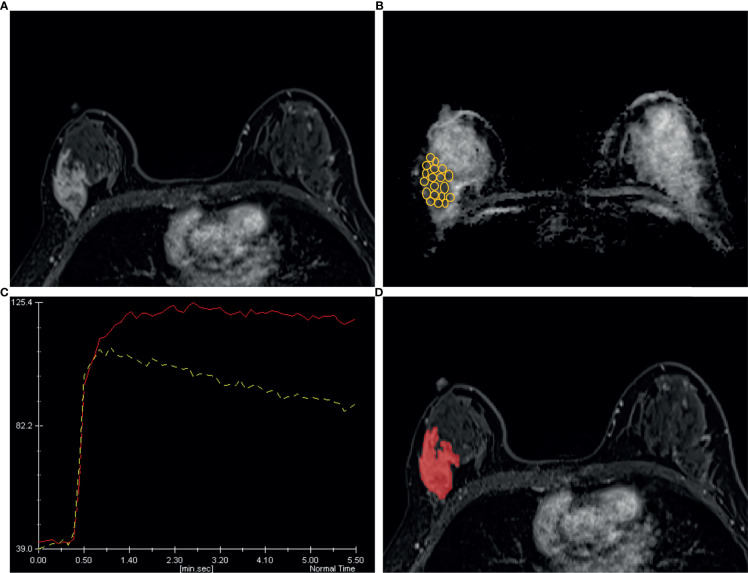
A 44 years old woman diagnosed as BIRADS 4b preoperatively by radiologists and confirmed as invasive ductal carcinoma by operation. **(A)** Axial dynamic contrast-enhancement images obtained in the very early phase (about 90 seconds) show a non-mass enhancement lesion with segmental distribution in the right breast. **(B)** The ADC map shows the minimum ADC value of the ROIs is 745 ×10^−6^ mm^2^/s. **(C)** After drawing the TIC curves for all ROIs at the brightest part on each slice, the high-level TIC curve type of this lesion is washout type. **(D)** Using the ITK-SNAP software, the whole lesion was segmented. Finally, the logistic regression equation of the combined model for this lesion was calculated as 0.989, which was higher than the cut-off value 0.772 and adjudicated as malignant lesion, consistent with the pathological results.

**Figures 6** and **7** show two cases in detail.

### 3.4 Specificity Changes

Considering the low specificity in the conventional clinical analysis, we conducted an analysis for the false positive (FP) lesions (n=33) and the true negative (TN) lesions (n=45) on the basis of the conventional clinical analysis in the whole cohort (78 benign NMEs). The results showed that compared to the TN lesions, the FP lesions had a significant larger proportion of moderate or marked BPE (P=0.004), plateau or washout type of TIC (P<0.001), and positive MIP sign (P<0.001). Of the 33 FP NMEs, 30 (90.9%) lesions were confirmed as adenosis, and the other 3 lesions were chronic inflammation. In addition, 21 of 33 (63.6%) FP lesions were categorized as malignancy applying the final combined model.

## 4 Discussion

In this study, we developed a clinical model that consisted of clinical characteristics, morphologic lesion assessments, the ALN status, TIC assessments on DCE-MRI, and minimum ADC values on DWI to differentiate benign and malignant NMEs. This model showed high sensitivity and low specificity in both the training (0.942, 0.589) and validation (0.940, 0.545) cohorts. To investigate the added value of the radiomics signature for NME differentiation, we added radiomics features derived from early phase DCE-MRI to the clinical model and built the combined model. The combined model achieved a higher specificity in the training (0.839) and validation (0.864) cohorts.

For the morphologic analysis, we used early phase images after contrast agent injection for NME evaluations because NMEs can be affected and obscured by more pronounced BPEs on the delayed phase images (29). Remarkably, although morphologic assessments, including distribution and internal enhancement patterns, were reported effective in previous studies (13–15), our study demonstrated that these morphologic features were not independently associated with NME differentiation, which is consistent with the results of a study by Naoko Mori et al. (10). Conversely, this lack of an independent association with morphologic features could be explained by decision-making pitfalls caused by the subjective judgment of visual examinations and by the variance of morphologic proportions contained in different study cohorts. In China, this can happen because the national breast cancer screening program is largely lacking compared with other countries; therefore, the lesions in the cohort of our study had larger sizes and a higher proportion of regional distributions and heterogeneous enhancement patterns. Thus, considering the potential role and subjective nature of morphologic assessments, we drew ROIs covering the whole lesion in each image plane and investigated the performance of the radiomics signatures alone, achieving a high sensitivity (82.7%) and specificity (80.4%). Of the six selected radiomics features, the surface area to volume ratio was given a maximum negative correlation (-0.594); lower ratios indicated a greater likelihood of NME malignancy, which is hard to identify with the human eye. Overall, these results indicated an important role for morphologic assessments in differentiating benign and malignant NMEs. However, it also indicated that histological patterns enrolled in the study may impact on the sensitivity and specificity of the model. The number of lesions in this study is relatively small, and further research should be undertaken in a large cohort to investigate the impact of different histological patterns on the differentiation performance of the model.

A previous study observed that minimum ADC values potentially suggested the presence of an invasive component in ductal carcinoma *in situ* (DCIS) (30). In our study, we applied the same approach for malignant component detection. To perform this approach, we assumed that the area with minimum ADC values corresponded to the region with the highest tumor cell density, reflecting malignancy. However, we demonstrated that malignant lesions had significantly lower minimum ADC values than benign lesions. The multivariate analysis indicated that the minimum ADC value was not an independent factor for the discrimination of benign and malignant lesions, suggesting a limited role for DWI. These results are consistent with those of some recent studies (9, 31).

Naoko Mori et al. reported that kinetic assessments might be more important than the morphologic assessments in differentiating benign from malignant NMEs on the ultrafast DCE-MRI (10). In this study, we employed a similar ultrafast DCE-MRI approach and achieved similar results. Comparatively, malignant lesions tended to have more neovascularization (32). Thus, it is reasonable to set the ROI on the brightest areas of the images during the very early phase after contrast injection to obtain TIC curves. The selection of higher TIC curve types could provide greater detection of malignant components in the lesion enhancements. The TIC type alone gave a higher sensitivity (94.2%) and lower specificity (58.9%) for NME differentiation.

Our results showed that MR-reported ALN alone offered a higher specificity (92.9%) and lower sensitivity (36.6%) than conventional DCE-MRI assessments, which could be explained since less axillary lymphadenopathy was detected on the MRI images of most patients with malignant or benign lesions in this study. However, this situation was not consistent with what is seen in clinical practice.

The analysis of low specificity showed that moderate or marked BPE, plateau or washout TIC, and MIP positive status may be prone to yield false positive results for NMEs in the conventional clinical analysis. It further indicated the difficulty and complexity of differentiation in clinical practice. Finally, the combined model of clinical features with added radiomics signature features improved the specificity in both the training (0.839) and validation cohorts (0.864). Given the comparable proportion of benign and malignant lesions and the good agreement between observers, the improved performance indicated that the radiomics signature was robust for the differentiation of benign and malignant NME lesions. The nomogram was primarily used to improve personalized diagnostics. The results of our study might suggest that additional radiomics signatures could help improve the specificity of differentiating benign and malignant NME lesions and avoid unnecessary biopsies. However, further studies with larger sample sizes are needed.

There were several limitations in our study. A primary limitation was the retrospective nature of the analysis, making potential selection bias difficult to avoid. Second, most of the patients in our hospital underwent breast MRI scans for two possible indications; preoperative staging for known breast cancer and further scanning for suspicious lesions in high-risk patients. Thus, the proportion of malignant lesions in our cohort was high, and there was a difference in the malignant/benign ratio between the training and validation cohorts. Third, the morphologic assessments and parameter measurements were accomplished by two radiologists using a consensus, and further research is needed to validate the repeatability of inter- and intra-observer. Fourth, the maximal diameters and morphologic assessments were recorded in the early phase to avoid being affected by BPEs; thus, some lesions with progressive enhancements might not have been evaluated accurately. Optimal timing needs to be determined in future studies.

In conclusion, the clinical multivariate regression analysis indicated that TIC patterns and ALN status were independent factors for the differentiation of benign and malignant NME lesions. Our results demonstrated that a radiomics nomogram combining clinical factors with radiomics signatures derived from early phase DCE-MRI could achieve high sensitivity and specificity for NME differentiation. Additional radiomics signatures could be used to improve specificity and avoid unnecessary biopsies. We believe that our model may not substitute but could improve conventional diagnostic workflow. However, a more extensive analysis with large samples is needed.

## Data Availability Statement

The raw data supporting the conclusions of this article will be made available by the authors, without undue reservation.

## Ethics Statement

The studies involving human participants were reviewed and approved by The ethics committee of Tongji Hospital. Written informed consent for participation was not required for this study in accordance with the national legislation and the institutional requirements.

## Author Contributions

YL, ZY, TA, and LX participated in the conception and design of the study. YL, YQ, and CT collected the clinical and imaging data. YL and WL performed the statistical analyses. YL, ZY, XY, YG, TA, and LX coordinated, drafted, revised and finalized the manuscript. All authors contributed to the article and approved the submitted version.

## Conflict of Interest

Author XY and YG were employed by the Siemens Healthcare company. WL was employed by Julei Technology Company.

The remaining authors declare that the research was conducted in the absence of any commercial or financial relationships that could be construed as a potential conflict of interest.

## Publisher’s Note

All claims expressed in this article are solely those of the authors and do not necessarily represent those of their affiliated organizations, or those of the publisher, the editors and the reviewers. Any product that may be evaluated in this article, or claim that may be made by its manufacturer, is not guaranteed or endorsed by the publisher.
